# Cognitive Impairment in Secondary Progressive Multiple Sclerosis: Effect of Disease Duration, Age, and Progressive Phenotype

**DOI:** 10.3390/brainsci12020183

**Published:** 2022-01-29

**Authors:** Bruno Brochet, Pierre Clavelou, Gilles Defer, Jérôme De Seze, Céline Louapre, Eloi Magnin, Aurélie Ruet, Catherine Thomas-Anterion, Patrick Vermersch

**Affiliations:** 1 Neurocentre Magendie Inserm U 1215, Université de Bordeaux, 146 rue de Léo Saignat, 33077 Bordeaux, France; 2 CRC-SEP, Hôpital Gabriel Montpied, CHU de Clermont-Ferrand, 58 Rue Montalembert, 63003 Clermont-Ferrand, France; pclavelou@chu-clermontferrand.fr; 3 CRC-SEP, Service de Neurologie, CHU de Caen, Avenue de la côte de Nacre, 14033 Caen, France; defer-gi@chu-caen.fr; 4 CRC-SEP, CHU Strasbourg, Hôpital Hautepierre, 1 Avenue Molière, 67098 Strasbourg, France; jerome.deseze@chru-strasbourg.fr; 5 Sorbonne University, Paris Brain Institute—ICM, Assistance Publique Hôpitaux de Paris, Inserm, CNRS, Hôpital de la Pitié Salpêtrière, CIC Neurosciences, 75013, Paris, France; celine.louapre@aphp.fr; 6 Service de Neurologie, Hôpital Jean Minoz, 1-3 Boulevard Alexandre Fleming, 25000 Besançon, France; eloi.magnin@laposte.net; 7 Neurocentre Magendie, INSERM U 1215, Université de Bordeaux, Service de Neurologie, CHU de Bordeaux, Hôpital Pellegrin, Place Amélie Raba Léon, 33076 Bordeaux, France; aurelie.ruet@chu-bordeaux.fr; 8 Centre Médical, 75 Rue Bataille, 69008 Lyon, France; c.thomas-anterion@orange.fr; 9 Inserm U1172—Lille Neuroscience et Cognition, Université de Lille, CRCR SEP, CHU Lille, FHU Precise, 59000 Lille, France; patrick.vermersch@univ-lille.fr

**Keywords:** multiple sclerosis, cognition, secondary progressive MS, neuropsychology

## Abstract

Background: Cognitive deficits are common in multiple sclerosis (MS) and affect patients at all stages of the disease, regardless of phenotype. Aims: This literature review focuses the cognitive deficits observed in secondary progressive MS (SPMS). It is mainly based on studies that compared the frequency and main characteristics of cognitive deficits in SPMS with other phenotypes. Methods: A bibliographic search was carried out using the PubMed database with the following keywords: multiple sclerosis, secondary-progressive, cognition. Results: Thirteen studies were initially selected that were published in English, reporting the neuropsychological data of a sample of at least 30 patients with SPMS, comparing them with patients with other phenotypes. Studies suggest that there is an association between the duration of the disease and the frequency and extent of the cognitive disorders. Studies also showed that the SP form is associated with an increased frequency of cognitive impairment and with an increased severity as compared to relapsing-remitting MS (RRMS). Compared to RRMS, progressive forms of MS are associated with more severe impairment in certain cognitive areas, such as episodic verbal memory, information processing speed, working memory, or verbal fluency. Two studies showed that cognitive performances decline overtime in SPMS. Conclusion: Cognitive disorders are more frequent and more severe in the SP form than in relapsing course of MS. The profile of cognitive impairment encountered in the SP form also appears to be different from those found in the other phenotypes.

## 1. Introduction

About 85% of people with multiple sclerosis (MS) begin their disease with a relapsing phenotype (relapsing-remitting MS (RRMS)). Patients with RRMS have acute exacerbations (relapses) with or without sequelae. An increasing number of disease-modifying drugs are available to treat patients with relapsing-remitting disease, preventing relapses. However, despite these treatments, a proportion of patients with RRMS evolves to secondary progressive MS (SPMS) over variable periods of time. This progressive stage is characterized by a continuous worsening of the disability, independent of additional new exacerbations, which may however still occur in some patients during this phase. SPMS, defined as a gradual worsening of neurological impairment and disability over several months regardless of any relapses, is usually diagnosed in patients with progressive worsening of the scores of the EDSS scale (Expanded Disability Status Scale), which is mainly a reflection of the analysis of gait and motor deficits.

Cognitive deficits are common in MS and affect patients at all stages of the disease—including the early stage [1]—and involve all phenotypes [2]. Epidemiological data collectively show that the frequency of cognitive deficits increases with disease. As SPMS follows RRMS, it is generally thought that the frequency of cognitive deficits is higher in that form than during the RR stage [2]. It has also been shown that in the primary progressive (PP) forms, cognitive disorders were more frequent, affected more cognitive domains and functions, and were more severe than in the RR forms, taking into account differences in age, sex, and duration of the disease [3]. This suggests that a higher frequency of cognitive impairment in SP forms may be linked not only to a longer duration of disease but also to the progressive phenotype. Methodological issues must be considered when comparing different studies. Firstly, neuropsychological tests vary widely between studies. The number of tests, the investigated areas, and the psychometric properties of the tests can affect the results. Secondly, the definition of cognitive impairment can also vary, for example, the number of abnormal scores required to define cognitive impairment, and the different statistical thresholds used. Thirdly, a recruitment bias can lead to a more systematic assessment of a given population in relation to the primary objective of these studies and the predefined inclusion criteria.

## 2. Methods

**Figure 1:**
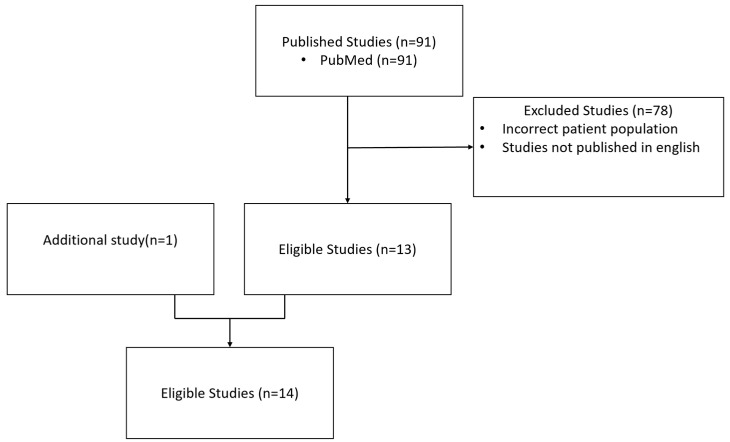
Flow chart of included studies.

A bibliographic search was carried out using the PubMed database with the following keywords: multiple sclerosis, secondary-progressive, and cognition (Figure 1). Of the 91 articles found, 13 studies were initially selected that were published in English, reporting the neuropsychological data of a sample of at least 30 patients with SPMS, and comparing them with patients with other phenotypes. A study was added because it reported the evolving data of a sample of patients with SPMS although not including the initial neuropsychological data [4]. Table 1 summarizes the characteristics of the main studies corresponding to these criteria and including a group of healthy controls. Some other studies did not include a control group of healthy subjects and are described in Table 2. A descriptive analysis of the selected studies was performed by noting relevant results to answer the following questions: (1) Is there an association between cognitive impairment frequency and disease duration; (2) is there an association between cognitive impairment frequency and the SP phenotype; (3) is there an association between cognitive impairment profile and the SP phenotype; and (4) what is the evolution of the cognitive impairment in the SP phenotype?

Other studies describing the evolution of other clinical phenotypes are also briefly discussed [5,6].

## 3. Results

### 3.1. Associations between Cognitive Disorders and Disease Duration

Two types of studies can investigate the link between cognitive disorders and disease duration: cross-sectional studies and longitudinal studies. The cross-sectional study conducted by Achiron et al. [7] was performed in a large sample of patients (1500) whose data were compared to normal values from 1569 healthy subjects. The authors used a battery of computerized cognitive tests. In this study, several phenotypes were represented, with 200 patients with clinically isolated syndrome (CIS), 1173 patients with RRMS, 100 patients with SPMS, and 27 patients with primary progressive MS (PPMS). Regardless of the definition of cognitive impairment of at least two standard deviations from the normal value for the overall cognitive score used (mean scores on different tests) or of at least one standard deviation, there was a significant increase in the frequency of cognitive impairment as a function of disease duration. Indeed, five years after the onset of the disease, 20.9% of patients had a cognitive decline defined by cognitive scores below one standard deviation, and 6% had a severe cognitive decline defined by cognitive scores below two standard deviations. The rates at 10 years were 29.3% and 9.0%, respectively.

In a multicentre study, Ruano et al. [8] also studied a large patient population (1040) including 167 patients with CIS, 759 with RRMS, 74 with SPMS, and 40 with PPMS. The authors used Rao’s Brief Repeatable Battery (BRB) as well as Stroop’s test, using normative data published for the Italian population. In the logistic regression analysis, there was a significant association (OR = 1.68, *p* < 0.001) between cognitive impairment and disease duration of more than 10 years (univariate regression analysis). In contrast, in multivariate analyses that specifically included age and phenotype, the association with disease duration was no longer significant: the two determinants of cognitive impairment were physical disability measured by the EDSS and the patient’s age despite the existence in some studies of a correlation between age and duration of illness.

Among the few longitudinal studies examining cognitive disorders in MS, the study by Amato et al. [5] compared the evolution of a population of patients recruited at an early stage of MS and followed for 10 years with that of a control group of healthy subjects assessed on the same cognitive battery [5]. Of the 50 patients initially recruited, 74% had no cognitive impairment (0 to 2 failed subtests) on initial assessment, while 8% had moderate impairment (failed 3 to 5 subtests), and 18% had severe impairment (more than 5 failed tests). At 10-year follow-up including 45 patients and 65 controls, the percentage of patients without significant cognitive impairment was 44%; 34% had a moderate cognitive impairment, and 22% had severe impairment. These results illustrate the increasing frequency of cognitive disorders over time and the extent of cognitive impairment in terms of number of tests and areas affected. However, no association with the phenotype has been demonstrated from these studies.

Another more recent study reported the evolution over six years of a group of 42 RRMS patients and 30 healthy subjects [6]. This study confirmed an increase in the frequency of cognitive impairment over time and an increase in the number of areas affected. This partly explains the higher frequency of cognitive disorders in patients with MS, in which the duration of the disease is generally longer.

These different data therefore suggest that there is an association between, on the one hand, the duration of the disease and, on the other, the frequency and extent of the cognitive disorders.

### 3.2. Associations between Cognitive Disorders and MS Phenotypes

In the cross-sectional study by Achiron et al. [7], the severity of the overall cognitive score was greater for the group of patients with SPMS than for the group of patients with CIS or RRMS (81.3 versus 92.1, *p* < 0.0001 and 90.6, *p* < 0.0001, respectively).

In a study aiming to validate the BRB battery [9], the authors compared 37 patients with CIS, 65 with RRMS, 31 with the SP form, and 35 with the PP form. They showed that patients with CIS and RRMS had a significant 90% reduction in the risk of cognitive disorders than those with SP and PP forms (OR: 0.10, 95% CI: 0.04–0.25 *p* < 0.001), taking into account age, sex, disease duration, and disability (OR: 0.10, 95% CI: 0.04–0.25 *p* < 0.001). The percentages of patients affected in these different phenotypes were, however not indicated.

In the multicentre study by Ruano [8], the percentage of cognitively affected patients, i.e., affected in at least two areas, was much higher in the SP form (79.4%) than in the CIS (34.5%) and RR (44.5%) forms. The question therefore arises as to whether this higher frequency and severity in the SP form is due to a longer disease duration or to the phenotype itself or both. This study [8] is in favour of a progressive phenotype effect, as there was a significant difference between the SP and CIS groups in the logistic regression analyses with cognitive deficit as a dependent variable, which included the phenotype in addition to age, EDSS, duration of illness, level of education, and gender. In this study, there was a significant difference in the same analysis between the PP group and the CIS group in favour of a progressive phenotype effect.

Several studies provide relevant information by comparing patients with the SP form to patients with RR forms with a comparable disease duration. These are patients with RRMS who did not evolve to SPMS after many years and who probably have milder forms of the disease.

The study by Smestad et al. [10], which included 123 patients with MS for more than 30 years (mean 34.5), is informative in this regard. This was a population-based study of 72 patients with SPMS, 36 with RRMS, and 14 with PPMS. Overall, across the population, there was significant cognitive impairment with substandard scores on most of the tests used. The duration of illness was not different on average between cognitively impaired patients (48% of patients, *n* = 40) and cognitively unaffected or for the severity of the handicap measured by EDSS, which was probably lower in the RR form than in the SP form, but that information was not provided. Logistic regression analysis with cognitive impairment as a dependent variable showed a significant association with the phenotype, the risk being higher in SP forms than in RR forms, with an odds ratio of 2.74 (95% CI: 1.01–7.44, *p* = 0.049) regardless of patient age. This study therefore suggests that the phenotype has an effect irrespective of age, which is linked to the duration of the disease.

The study carried out in the Auvergne MS network by Planche et al. [11] also compared patients with an SP form (*n* = 37) to patients with a late RRMS (*n* = 41). A smaller sample of 23 patients with PPMS was also studied. Disease duration and age were not different between the SPMS and LRRMS groups. In contrast, as expected, the level of disability (EDSS) was higher in the SPMS group. The percentage of cognitively impaired patients in at least two domains was significantly higher in the SPMS group compared with the late RRMS group after adjusting for age, sex, educational level, duration of disease, and disability measured by EDSS. These results are again in favour of an effect of the phenotype rather than the severity of the disease.

In the French validation study of the MACFIMS (Minimal Assessment of Cognitive Function in MS) battery, the frequency of cognitive impairment was twice as high in the SPMS group (*n* = 46) than in the RRMS group (*n* = 43). The two groups were not compared directly with each other but with distinct groups of control subjects matched to each of the groups by age, sex, and level of education [12].

In light of these data, it seems that SP form is associated with an increased frequency of cognitive impairment and with an increased severity.

### 3.3. Association between Cognitive Impairment Profile and MS Phenotype

In a study carried out in Lorraine, Brissart et al. [13] compared a group of 36 patients with SPMS to 31 patients with early RRMS, 37 patients with late RRMS, 24 with PPMS, and a group of 63 healthy subjects. The late RRMS and SPMS groups had comparable disease duration but a higher level of disability in the SPMS group. The tests for which there was a significant difference between the patients’ scores with early RRMS and SPMS and late RRMS were the same. These were tests for information processing speed (SDMT, Symbol Digit Modalities Test) and the verbal episodic memory tests. This study suggested the presence of more severe involvement in these domains in the SP forms. There was no significant difference between the PP and SP forms.

The study by Huijbregts et al. [14] compared 108 patients with RRMS, 71 patients with SPMS, 55 with PPMS, and 67 healthy control subjects. Patients with SPMS were older, had greater disability, and longer disease duration than patients with RRMS; PPMS patients were older and had greater disability than SPMS patients. The severity of the impairment was greater in the SPMS forms than in the RRMS forms for memory tests (SRT, Selective Reminding Test; and SPART, Spatial Recall Test), for information processing speed (SDMT, Symbol Digit Modalities Test), and for working memory (PASAT, Paced Auditory Serial Addition Task). The magnitude of the effect between these two clinical forms was highest for the SDMT test. The performance of SPMS patients was poorer than those with PPMS when the tasks required working memory at a higher level, except for those where the information processing speed played a relatively important role (SDMT, PASAT).

Rosti-Otajärvi et al. [15] compared 138 patients with RRMS, 32 patients with SPMS, and 26 with PPMS, all with cognitive impairment. They observed a difference in information processing speed (SDMT, *p* < 0.001), which was more severe in the progressive forms, while the difference in other cognitive domains (visual memory, lexical fluency, and working memory) was not significant, probably due to insufficient statistical power. When the progressive forms were assessed separately, patients with PPMS had lower scores than those with SPMS or RRMS in the domains of verbal memory (BSRT/CLTR, Buschke Selective Reminding Test/Consistent Long-Term Retrieval; BSRT/LTS, Buschke Selective Reminding Test/Long-Term Storage), working memory and processing speed (SDMT), and verbal fluency (COWAT, Controlled Oral Word Association Test).

A recent multicentre study conducted in Germany [16] used the BICAMS (Brief International Cognitive Assessment for Multiple Sclerosis) battery in a large sample of 978 patients with RRMS, 87 patients with SPMS, and 29 patients with PPMS. Progressive forms were associated with lower processing speed scores (SDMT), SPMS forms with lower visual learning scores (BVMT-R, Brief Visuospatial Memory Test Revised), and PPMS forms had a lower score on the verbal episodic memory test (VLMT, *Verbaler Lern- und Merkfaehigkeitstest*).

However, these studies did not include detailed tests of executive functions apart from measuring verbal fluency. In the initial validation study of the MACFIMS [17] battery, a more precise evaluation of executive functions was used (the DKEFS-ST classification test, Delis Kaplan Executive Function System Sorting Test). This study included 78 patients with SPMS and 200 patients with RRMS. The principal component analysis (PCA) did not show any difference in the distribution of profiles between the RR and SP forms for components derived from the neuropsychological scores, namely impairment in information processing speed and of working memory for the first component and impairment of memory and executive function for the second component.

In a study that compared 43 patients with RRMS and 45 with SPMS, Kizlaitiéné et al. [18] observed significant differences in several cognitive domains, using ANCOVA with adjustment for age and level of education but not for EDSS. In comparison to RR forms, patients with MS forms had higher scores for the TMT (Trail Making Test, evaluating flexibility) but lower for the DSST (Digit Symbol Substitution Test, evaluating the speed of psychomotor reaction and attention), the CATflT (Category Fluency Test, evaluating verbal fluency), the RAVLT 1-5 SUM (sum of the recalled words in the first 5 attempts to learn the word list of the Rey Auditory Verbal Learning Test, evaluating verbal memory and verbal learning), and the Story (Short Story Test, assessing logical memory).

In a study of 311 patients with MS but no healthy subjects, Matias-Guiu et al. [19] compared 236 patients with RRMS, 52 with SPMS, and 23 with PPMS. Using a battery including several tests of executive functions, they were able to show by PCA that cognitive impairment was more severe in the SP and PP forms than in the RR forms for this executive domain (*p* = 0.014) as well as for verbal memory (*p* = 0.01).

In the multicentre study by Ruano [8], several logistic regression analyses were carried out, with the percentages of patients impaired in the main cognitive domains as dependent variable; these main cognitive domains included verbal learning, visuospatial learning, speed of treatment of the information, and executive functions. Executive functions were the only cognitive domain in which the impairment was associated with MS phenotype after adjusting for other variables. This can be compared with the results observed in the PP and the RR forms. The adjusted OR for executive functions was 1.95 (95% CI: 1.29–2.96) for the RRMS vs. CIS comparison; 2.61 (95% CI: 1.25–5.44) for the RRMS vs. SPMS comparison; and 15.02 (95% CI: 1.85–122.12) for the RRMS vs. PPMS comparison.

In a study including 60 patients with RRMS, 41 PPMS patients, and two corresponding groups of controls matched by age, sex, and level of education, Ruet et al. [3] observed a difference in frequency of cognitive impairment for episodic memory and executive functions between the two phenotypes as well as a difference in effect size for many neuropsychological tests (in particular executive functions, episodic verbal memory, working memory, and the speed of information processing). In this study, disease duration for PPMS and RRMS forms was not significantly different. After ANCOVA analysis adjusted for EDSS, differences in frequency of cognitive impairment and effect size remained significant.

Analysis of the literature suggests that compared to RR forms, progressive forms of MS are associated with more severe impairment in certain cognitive areas, such as episodic verbal memory, information processing speed, working memory, or verbal fluency.

### 3.4. The Evolution of Cognitive Disorders over Disease Course

Few studies have compared the “annual mean cognitive change” using a complex algorithm accommodating the effect of practice according to different phenotypes. In a study focusing on MRI outcomes, Eijlers et al. [4] compared these annual mean changes in 182 patients with RRMS, 33 patients with SPMS, 19 patients with PPMS, and 60 control subjects over a period of five years. Patients with the SP and PP forms were more likely to have cognitive decline (55% and 53%, respectively) compared with RRMS patients (21%). Huijbregts et al. [20] followed the progress of 30 patients with SPMS over two years and showed that, unlike healthy subjects, there was no improvement in information processing speed scores and working memory (PASAT and SDMT), suggestive of degradation over time in at least these two areas. The authors conclude that for tasks that place high demands on these neuropsychological domains, the lack of improvement with learning indicates a short-term manifestation of cognitive decline.

It therefore seems from these two studies that in patients with MS, cognitive impairment worsens over time, particularly in the progressive forms.

## 4. Discussion

This review of the literature shows that the number of studies devoted to cognitive disorders in the SPMS form remains rather limited, while it also confirms that cognitive disorders are more frequent than in the RRMS form.

This review addressed the question of the respective roles of the duration of the disease and the phenotype in this high frequency. The available data reviewed here seem to show that both elements play a role. The various longitudinal and cross-sectional studies on the influence of disease duration have shown that the frequency of cognitive impairment increases with disease duration. This suggests that the higher frequency observed in the MS forms is partly related to this duration. It has also been shown that the duration of the disease also increases the number of cognitive domains affected. However, a non-specific role of age, which is, of course, very much related to the duration of the disease, cannot be completely ruled out.

Studies on the role of the phenotype show, however, that the influence of the duration of the disease is not the only factor that explains the increase in the frequency and severity of cognitive disorders in MS forms. There also seems to be an important influence of the progressive phenotype. Indeed, various studies have shown an independent effect of the phenotype on the frequency, and moreover, the high frequency of cognitive disorders in the PP forms and their great severity are consistent with this. This can be explained by the neuro-pathological mechanisms specific to progressive forms, such as the important involvement of the cortex and the importance of axonal involvement [21].

Finally, very few studies have looked at the longitudinal evolution of cognitive disorders in MS. The available data suggest a marked evolution comparable to that of PP forms and greater than that of RR forms.

All these results suggest some important practical conclusions for the management of patients. Early therapeutic management of patients at the RR stage could reduce the risk of progression to the progressive form and thus to a higher frequency and severity of cognitive disorders. On the other hand, in patients with the MS form, it is necessary to identify these disorders by means of appropriate neuropsychological assessments in order to propose an adaptation of the therapeutic management.

## 5. Conclusions

This literature review shows that there are different parameters that appear to influence cognitive impairment; cognitive impairment becomes more frequent and worsens over time, and the MS phenotype plays a role. In particular, studies suggest that compared with RR or CIS forms, SP forms have more frequent and more severe cognitive impairment. The profile of cognitive impairment is also different in the progressive forms in the various studies reviewed: greater impairment of executive functions, information processing speed, verbal memory, and working memory. 

## Figures and Tables

**Table 1:** Controlled studies about cognition in secondary progressive multiple sclerosis.

Author	Number of Centres	Number of Patients				Comments and Main Evaluation Criteria	Primary Results
		SPMS (*N*)	PPMS (*N*)	RRMS (*N*)	Control Subjects (*N*)		
*Benedict, 2006*	1	78	7	200	56	+6 patients with PPMS (primary progressive) The aim of the study was to assess the validity of MACFIMS as a tool for detecting cognitive impairment in patients with MS.	The 7 neuropsychological evaluation tests used by the MACFIMS enabled identification of healthy individuals and patients with MS and to distinguish patients with the RR form from those with SP form, with an effect size varying from medium to very high.
*Brissart, 2013*	1	36	24	78	63	The main objective was to describe cognitive impairment in the early stages of MS; the secondary objective was to compare cognitive performance according to MS phenotypes.	Compared to RR forms, patients with SP forms had more severe involvement on SDMT and RAVLT. There was no significant difference between the PP and SP forms.
*Eijlers, 2018*	1	33	19	182	60	The aim of the study was to identify MRI parameters and demographic and/or clinical data that could be predictive of cognitive decline during 5-year follow-up with BRB.	66/234 patients (28%) presented a cognitive decline during follow-up, particularly in the progressive forms: 18/33 patients with SPMS (55%), 10/19 patients with PPMS (53%), and 38/182 patients with RRMS (21%).
*Huijbregt, 2004 & 2006*	1	71	55	108	67	The aim of the study was to explore the cognitive functions of patients with MS and to assess the impact of the different phenotypes of the disease. The 2-year follow-up is presented in the 2006 study. Assessment by BRB.	The severity of the impairment was greater in the SPMS than in the RRMS forms for memory tests (SRT and SPART) for information processing speed (SDMT) and for working memory (PASAT). The severity of the impairment was greater in the SPMS forms than in the RRMS forms for memory tests (SRT, Selective Reminding Test; and SPART) for the speed of information processing (SDMT, Symbol Digit Modalities Test) and for working memory (PASAT, Paced Auditory Serial Addition Task).
*Maubeuge, 2020*	15	46	45	43	276	The aim of the study was to validate the French version of the MACFIMS.	The cognitive deficit concerned 33.7% of patients with MS: 42.9% in the SPMS group, 35.3% in the PPMS group, and 18.8% in the RRMS group.

BRB, Brief Repeatable Battery; MACFIMS, Minimal Assessment of Cognitive Function in MS; PASAT, Paced Auditory Serial Addition Task; RAVLT, Rey Auditory Verbal Learning Test; SDMT, Symbol Digit Modalities test; SPART, Spatial Recall Test; SRT, Selective Reminding Test.

**Table 2:** Other studies about cognition in secondary progressive multiple sclerosis.

Author	Number of Centres	Number of Patients				Comments and Main Evaluation Criteria	Primary Results
		SPMS (*N*)	PPMS (*N*)	RRMR (*N*)	Control Subjects (*N*)		
*Achiron, 2013*	**1**	**100**	27	1173	Controls (1569 healthy subjects)	+200 patients with CIS The objective of the study was to evaluate the evolution of cognitive capacities in patients with MS via a battery of computerized tests evaluating verbal and non-verbal memory, executive functions, visuospatial perception, verbal function, attention, speed of information processing, and motor skills.	5 years after disease onset, 20.9% of patients had a cognitive decline of one standard deviation and 6% a severe cognitive decline of two standard deviations. The 10-year rates were 29.3% and 9.0%. The severity of the overall cognitive score was greater for the group of patients with SPMS than in the group of patients with CIS or RRMS (81.3 versus 92.1, *p* < 0.0001 and 90.6, *p* < 0.0001).
*Dackovic, 2016*	1	31	35	65	0	+37 patients with CIS. The aim of the study was to assess cognitive performance based on MS phenotypes using BRB.	58.9% of patients presented with cognitive impairment: 40.5% in the CIS group, 36.9% in the RRMS group, 96.8% in the SPMS group, and 85.7% in the PPMS group (before adjustment, percentages not specified after adjustment).
*Kizlaitiené, 2017*	1	45	-	43	0	The objective of the study was to identify a simple way to discriminate between RRMS and SPMS forms that is applicable in clinical practice, based on MRI data and evaluation of cognitive performance using a specific battery.	The study proposed a composite marker based on imagery and cognitive testing to discriminate between RRMS and SPMS.
*Matias-Guiu 2017*	1	52	23	236	Norms	The aim was to determine the frequency of cognitive deficits and the main cognitive domains affected and to identify the factors associated. The neuropsychological evaluation was carried out using a specific battery.	Cognitive decline identified in 41.5% of patients, significantly more frequent in patients with forms of SP and PP (*p* = 0.002). The mean scores for the items verbal memory and superior executive functions were higher in patients with RRMS than in patients with a progressive form.
*Planche, 2015*	1 + network	37	23	41	0	Retrospective analysis of cognitive tests (BRB or others) from a MS patient database to explore the distribution and frequency of different phenotypes.	63% of the patients had a significant cognitive decline (36.6% in the RRMS group, 86.1% in the SPMS group, and 73.9% in the PPMS group). Patients with SPMS had double the risk of cognitive decline compared with RRMS patients.
*Renner, 2020*	65	87	29	978	0	Multicentre study to characterize cognitive deficits and to identify predictive markers of cognitive decline using in particular the BICAMS battery.	Cognitive disorders were present in 28% of patients and more frequent in the progressive forms (SP: 45.9%, PP: 44.8%) than in the RR form (25.8%).
*Rosti-Otajarvi, 2014*	1	32	26	138	0	The aim of the study was to assess the extent to which cognitive complaints by patients with different MS phenotypes were associated with specific cognitive deficit profiles using the BRB.	A significant difference was noted between the progressive forms and the RR forms using the SDMT (37.7 vs. 44.9, *p* = 0.001).
*Ruano, 2017*	6	74	40	759	0	+167 patients with CIS. The aim of the study was to compare the prevalence and characteristics of cognitive impairment in a population of patients with MS, based on the BRB and the Stroop test.	The percentage of cognitively impaired patients was significantly higher in the SP form (79.4%) versus the CIS (34.5%) and the RR (44.5%) forms.In multivariate analysis, the determinants of cognitive decline were age and physical disability.
*Smestad, 2010*	1 + region	72	14	36	0	The aim of the study was to assess the evolution of cognitive abilities over 3 decades in patients with MS via a battery of specific neuropsychological tests.	After 30 years of MS, 48% of patients presented with cognitive decline. Logistic regression analysis with cognitive deficit as the dependent variable showed a significant association with the phenotype, the risk being higher in SPMS forms than in RRMS forms with an odds ratio of 2.74 (*p* = 0.049).

BICAMS, Brief International Cognitive Assessment for Multiple Sclerosis; BRB, Brief Repeatable Battery.

## Data Availability

Not applicable.
